# Clinical warning of hemophagocytic syndrome caused by Epstein-Barr virus

**DOI:** 10.1186/s13052-020-00949-7

**Published:** 2021-01-07

**Authors:** Jinjin Shi, Chu Chu, Min Yu, Dandan Zhang, Yuqin Li, Yujie Fan, Yixue Yu, Yali Luo, Weifang Zhou

**Affiliations:** Department of Infectious Disease, Children’s Hospital of Soochow University, Suzhou, 215003 China

**Keywords:** Infectious mononucleosis, Hemophagocytic syndrome, EBV DNA load, D-dimer

## Abstract

**Objectives:**

This study aimed to compare the clinical features and laboratory tests of infectious mononucleosis (IM) and hemophagocytic syndrome (HLH) caused by Epstein-Barr virus (EBV) in 1–3-year-old children and to explore the risk factor of HLH caused by EBV (EBV-HLH).

**Methods:**

The clinical data of 92 children with EBV infection admitted in our hospital from 2011 to 2019 were collected; 61 cases were diagnosed as EBV-IM, and 31 cases were diagnosed as EBV-HLH. The subjects’ clinical manifestations and laboratory tests were analyzed retrospectively.

**Results:**

Compared with EBV-IM patients, EBV-HLH patients had longer durations of fever, both before hospitalization and overall, and a higher probability of hepatomegaly. The levels of ALT, AST, LDH, TG, SF, D-Dimer and the plasma EBV DNA load of EBV-HLH patients were significantly higher than those of EBV-IM patients. The absolute values of CD3^+^, CD4^+^, CD8^+^, NK, and CD3-CD19^+^ cells and IgA and IgM levels of EBV-HLH patients were significantly lower than those of EBV-IM patients. The plasma EBV DNA load was positively correlated with the PT, TT, α-HBDH, AST, LDH, CK, Scr, BUN, UA, TG, and CRP levels in EBV-HLH patients, and the plasma EBV DNA load was positively correlated with the D-Dimer level in the EBV-IM patients. Among the 10 different potential markers, at the cut-off point of 1721.500 μg/L, the sensitivity and specificity of D-Dimer was 88.90 and 90.20%, respectively.

**Conclusion:**

The D-Dimer level may be a good prognostic indicator of EBV-HLH caused by EBV.

## Introduction

Epstein-Barr virus, also known as Human Herpes virus type IV, is a 172-kb double-stranded linear DNA virus. It was the first DNA virus found to be associated with human tumors, and EBV is generally latent for life after the initial infection. The infection rate of EBV in the population is > 95%, and most primary infections present as infectious mononucleosis (EBV-IM), with a self-limited disease course. In a subset of children infected with this virus, the disease progresses to EBV-related hemophagocytic syndrome (EBV-HLH), which has a mortality rate of over 50% [1]. For children with mild symptoms or in the early stage of the disease, they may fail to meet the diagnostic conditions after routine examinations, leading to missed diagnosis. We hope to find clues for early diagnosis of hemophagocytic syndrome and carry out intervention treatment early to improve the prognosis of children. Here, cases of EBV infection at Children’s Hospital of Soochow University from 2011 to 2019 were analyzed retrospectively and the clinical features and laboratory test results of cases of EBV-IM and EBV-HLH were compared to identify potential warning indexes of EBV-HLH.

## Materials and methods

### Research subjects

Studies at home and abroad have shown [2, 3] that hemophagocytic syndrome is more common in infants and young children, and we select children in early childhood to ensure their age comparability. The clinical data and patient characteristics of 92 children with EBV infection admitted to Children’s Hospital of Soochow University from 2011 to 2019 were retrospectively analyzed, including their sex, age, length of stay, routine blood analysis, lymphocyte subset counts, humoral immunity, and EBV DNA load. All patients had no previous history of recurrent infections, immunodeficiency or special medication.

### Diagnostic criteria

All cases of EBV-IM were diagnosed based on a combination of clinical symptoms and laboratory confirmation [4]. The clinically diagnosed cases were defined as those meeting any three of the six clinical indicators, along with the fourth laboratory indicators, described below; the laboratory confirmed cases were defined as those meeting any three of the six clinical indicators, along with at least one of the first three laboratory parameters described below. The six clinical parameters were: (1) fever; (2) pharyngillary tonsillitis; (3) cervical lymphadenopathy; (4) splenomegaly; (5) hepatomegaly; (6) eyelid edema. The four laboratory indicators were: (1) positive detection of EBV viral capsid antigens (VCA) IgM and EBV VCA IgG antibodies, but negative results for EBV nuclear antigen (NA) IgG antibody; (2) negative results for EBV VCA IgM antibody, but positive detection of EBV VCA IgG antibody and low-affinity; (3) an increase in the titer of EBV VCA IgG antibody of more than four times the original level in a second serum sample; (4) a proportion of heterotypic lymphocytes in the peripheral blood of ≥0.10 and/or lymphocytosis of ≥5.0 × 10^9^/L.

The diagnosis of EBV-HLH must meet the diagnostic criteria of HLH, with evidence of EBV infection.

Cases of EBV-HLH were diagnosed in children meeting five of the following eight diagnostic criteria [5]: (1) fever; (2) splenomegaly; (3) cytopenias affecting at least two of three lineages in the peripheral blood, defined as a hemoglobin < 90 g/L, platelets < 100 × 10^9^/L, and/or neutrophils < 1.0 × 10^9^/L; (4) hypertriglyceridemia and/or hypofibrinogenemia, defined as fasting triglycerides ≥3.0 mmol/L (≥2.65 g/L) and fibrinogen ≤1.5 g/L; (5) hemophagocytosis in bone marrow or spleen or lymph nodes and no evidence of malignancy; (6) low or absent natural killer (NK) cell activity (based on local laboratory reference); (7) ferritin ≥500 μg/L; (8) soluble CD25 (soluble IL-2 receptor) ≥2400 U/mL.

Diagnosis of EB virus infection [6]: (1) Serological antibody test prompts primary acute EBV infection (meets any of the following three indicators: positive detection of EBV CA IgM and EBV CA IgG antibodies, but negative results for EBV NA IgG antibody; negative results for EBV CA IgM antibody, but positive detection of EBV VCA IgG antibody and low-affinity; double serum EBV CA IgM antibody titer increased by more than four times) or active infection (serum EBV antibody titer abnormally increased, including EBV CA IgG antibody ≥1:640 or EBV EA IgG antibody ≥1: 160, positive detection of EBV CA IgA antibody and/or EBV EA IgA antibody); (2) molecular biology methods including PCR, in situ hybridization and Southern hybridization EBV-positive was detected from the patient’s serum, bone marrow, lymph nodes and other affected tissues, such as positive results for serum and/or plasma EBV-DNA, EBV-EBERs in situ hybridization or EBV-LMP1 immunohistochemical staining in the affected tissues.

### Experimental method

EBV DNA detection was performed as follows: 2 ml of patient venous blood was collected in the presence of the anticoagulant ethylene diamine tetraacetic acid (EDTA). The blood samples of all patients were collected within 2 h after admission. The EBV DNA load in the serum sample was then detected by real-time fluorescent polymerase chain reaction (PCR), using reagents produced by Hunan Shengxiang Biotechnology Co. LTD (Hunan, China). Samples were analyzed using a Roche Light Cycler 480 real-time fluorescent PCR machine, which was operated strictly in accordance with the reagent and instrument specifications.

### Statistical methods

Data were analyzed with SPSS version 24.0 (IBM Corp., Armonk, NY, USA). The measurement data conforming to the normal distribution were expressed by mean (SD), the measurement data not conforming to the normal distribution were expressed by median (IQR), and the Mann-Whitney U test was used for pairwise comparison between groups. A chi-square test was used for comparing numerical data, and a linear regression was used to analyze correlation. The inspection level was 0.05, and *p* < 0.05 was considered statistically significant. Receiver Operating Characteristic (ROC) curve analysis was used to evaluate the diagnostic value of each index, and *p* < 0.05 was considered statistically significant.

## Results

The clinical data and patient characteristics of 92 children with EBV infection admitted to Children’s Hospital of Soochow University from 2011 to 2019 were retrospectively analyzed. Based on the diagnostic criteria described above, the 92 children with EBV infection were divided into two groups: EBV-IM group: 61 children, 29 males and 32 females, with an average age of 1.75 (0.57) years, and EBV-HLH group: 31 children, 13 males and 18 females, with an average age of 1.77 (0.46) years. There was no significant difference in sex and age between the two groups (*p* < 0.05).

For the EBV-IM and EBV-HLH groups, the time with fever before hospitalization was 4.04 (1.990) days and 8.97 (5.672) days, peak body temperature was 39.00 (0.654) °C and 39.79 (0.569) °C, and duration of fever was 6.59 (4.660) days and 14.03 (6.631) days, respectively (all *p* < 0.001). In EBV-IM group, 32.70% of patients had hepatomegaly and 95.08% had superficial lymphadenopathy, compared with 70.97 and 38.71%, respectively, in EBV-HLH group (*p* = 0.001; < 0.001, respectively). There was no significant difference in splenomegaly between EBV-IM group (45.90%) and EBV-HLH group (64.52%) (*p* = 0.091).

Some of the children with EBV-IM had accompanying liver function damage, which was mainly mild. In contrast, all organs could be affected in the children with EBV-HLH. The laboratory indexes at the initial diagnosis were compared between the two groups. The data indicate that the levels of alanine aminotransferase (ALT), aspartate aminotransferase (AST), lactate dehydrogenase (LDH), triglyceride (TG), serum ferritin (SF), and D-Dimer of EBV-HLH group were all significantly higher than those of EBV-IM group (Table 1).

**Table 1:** Biochemical comparison between EBV-IM group and EBV-HLH group

	EBV-IM group	EBV-HLH group	Statistic value	*P* value
ALT(U/L)	53.000(30.500,103.800)	195.900(50.700,587.000)	Z = 3.783	< 0.001
AST(U/L)	57.700(39.700,88.000)	331.000(103.000,1045.000)	Z = 5.931	< 0.001
LDH(U/L)	547.100(499.000,669.000)	1228.900(839.000,1816.100)	Z = 5.885	< 0.001
TG (mmol/L)	1.740(0.887)	3.700(2.273)	t = −4.547	< 0.001
SF (ng/mL)	115.350(64.380,173.780)	1650.000(1316.600,11,675.900)	Z = 7.433	< 0.001
D-Dimer (ug/L)	878.000(489.000,1143.000)	4830.000(3331.000,12,000.000)	Z = 6.040	< 0.001

The absolute values of the CD3^+^, CD4^+^, CD8^+^, NK, CD3-CD19^+^ cell counts in EBV-HLH group were significantly lower than those in EBV-IM group. Additionally, the IgA and IgM levels in EBV-HLH group were also significantly lower than those in EBV-IM group. There was no significant difference between groups in the level of IgG (Table 2).

**Table 2:** Comparison of absolute count of peripheral blood lymphocytes and immunoglobulin levels between EBV-IM group and EBV-HLH group [M (P25, P75)]

Group	CD3+ (×10^9^/L)	CD4+ (× 10^9^/L)	CD8+ (× 10^9^/L)	NK (× 10^9^/L)	CD3-CD19+ (× 10^9^/L)	CD4+/CD8+ (× 10^9^/L)	IgA (g/L)	IgG (g/L)	IgM (g/L)
EBV-IM group	8.21 (4.98,11.63)	1.87 (1.19,3.00)	4.36 (3.00,6.77)	0.89 (0.62,1.92)	0.85 (0.43,1.29)	0.40 (0.30,0.60)	1.30 (0.92,1.92)	10.32 (8.63,12.52)	1.72 (1.31,2.08)
EBV-HLH group	0.93 (0.53,2.67)	0.43 (0.19,1.00)	0.55 (0.20,1.26)	0.10 (0.02,0.22)	0.15 (0.07,0.43)	0.90 (0.40,1.65)	0.48 (0.17,0.90)	6.97 (4.13,14.08)	0.57 (0.28,0.94)
Z value	−6.411	−5.763	− 6.290	− 6.468	− 5.401	3.856	− 5.496	−1.677	− 5.562
*P* value	< 0.001	< 0.001	< 0.001	< 0.001	< 0.001	< 0.001	< 0.001	0.094	< 0.001

The plasma EBV DNA load of EBV-HLH group was significantly higher than that of EBV-IM group (Table 3).

**Table 3:** Comparison of total blood and plasma EBV DNA load from peripheral blood between EBV-IM group and EBV-HLH group [M (P25, P75), copies/mL]

Group	EBV-IM group	EBV-HLH group	Z value	*P* value
Total blood EBV-DNA load	0.44(0.09,1.42)× 10^6^	2.96(0.32,20.55)×10^6^	3.006	0.003
Plasma EBV-DNA load	0.43(0.16,1.59)×10^4^	35.85(4.26,114.20)×10^4^	4.300	0.001

The plasma EBV DNA load was positively correlated with the levels of prothrombin time (PT), thrombin time (TT), a-hydroxybutyrate dehydrogenase (α-HBDH), AST, LDH, creatine kinase (CK), serum creatinine (Scr), blood urea nitrogen (BUN), uric acid (UA), TG, and C-reactive protein (CRP) in EBV-HLH group, and the plasma EBV DNA load was positively correlated with the level of D-Dimer in EBV-IM group (Table 4).

**Table 4:** Correlation between plasma EBV DNA load and various laboratory indicators in EBV-IM group and EBV-HLH group

	Plasma EBV-DNA Load of EBV-IM group		Plasma EBV-DNA Load of EBV-HLH group	
	r value	*P* value	r value	*P* value
D-Dimer (ug/L)	0.720	< 0.001	0.132	0.627
APTT(s)	−0.061	0.650	0.244	0.345
PT(s)	0.108	0.424	0.506	0.032
TT(s)	−0.091	0.501	0.812	< 0.001
α-HBDH(U/L)	0.169	0.227	0.981	< 0.001
ALT(U/L)	−0.058	0.674	0.386	0.113
AST(U/L)	−0.017	0.903	0.615	0.007
LDH(U/L)	−0.236	0.082	0.920	< 0.001
CK(U/L)	0.088	0.528	0.601	0.008
Scr (μmol/L)	0.220	0.107	0.834	< 0.001
BUN (mmol/L)	−0.087	0.527	0.821	< 0.001
UA (μmol/L)	0.090	0.512	0.878	< 0.001
TG (mmol/L)	−0.163	0.240	0.506	0.032
CRP (mg/L)	0.060	0.655	0.733	0.001

**Fig. 1:**
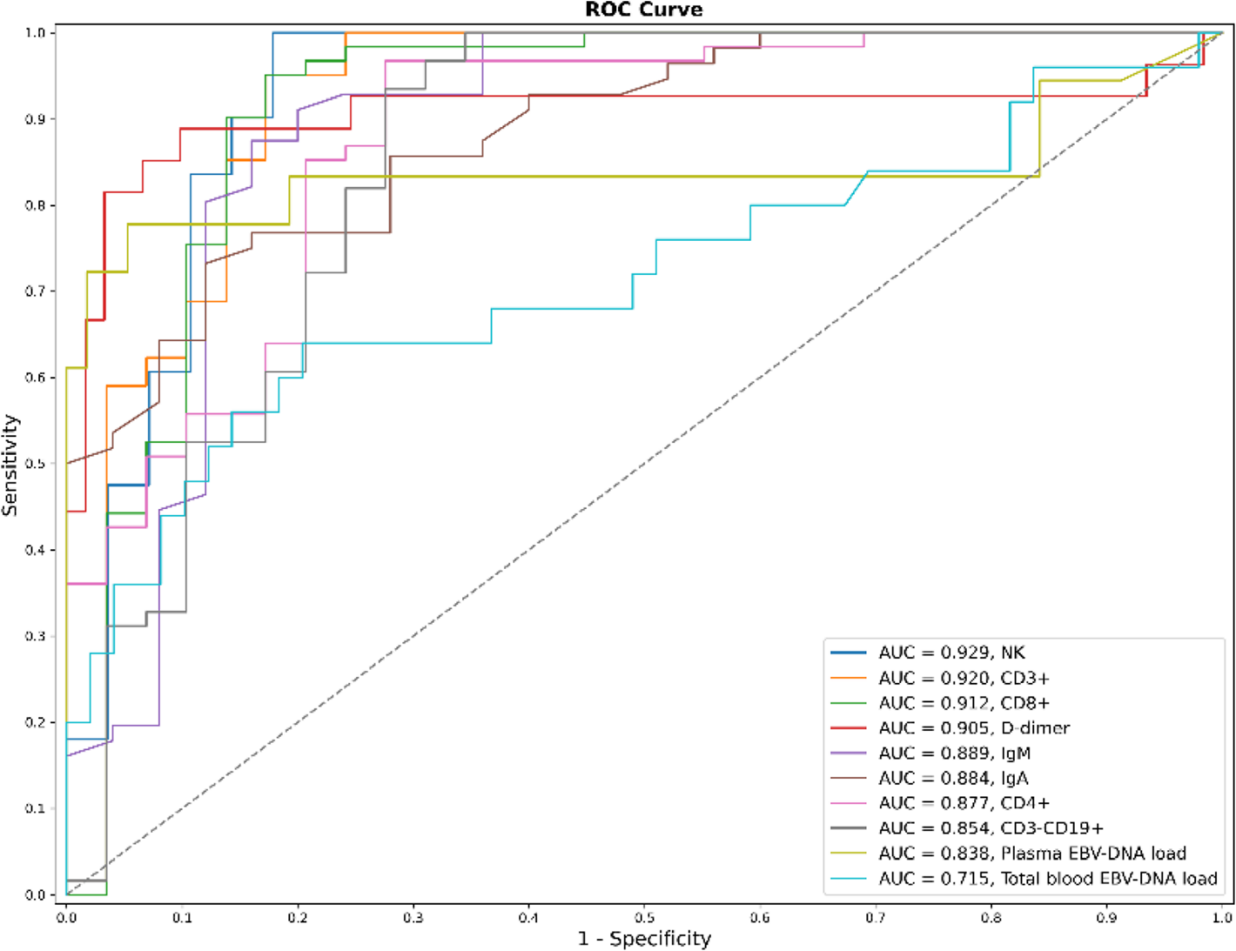
The ROC curve of EBV-IM and EBV-HLH

Among the ten markers identified here as being significantly different between groups, the D-Dimer level was the most valuable indicator for distinguishing between cases of EBV-IM and EBV-HLH. At the cut-off point of 1721.500 μg/L, the sensitivity and specificity of D-Dimer was 88.90 and 90.20%, respectively. Figure 1 shows the ROC curve of EBV-IM and EBV-HLH. The diagnostic efficacy indicators of each index for EBV-IM and EBV-HLH are shown in Table 5.

**Table 5:** The diagnostic efficacy indicators of each index for EBV-IM and EBV-HLH

	AUC	95%CI	*P* value	Cut-off point	sensitivity	specificity
IgA(g/L)	0.884	0.811–0.957	< 0.001	0.965^a^	88.00%	73.20%
IgM(g/L)	0.889	0.793–0.984	< 0.001	1.145^a^	84.00%	87.50%
CD3 + (×10^9^/L)	0.920	0.843–0.996	< 0.001	3.338^a^	82.80%	95.10%
CD4 + (×10^9^/L)	0.877	0.797–0.957	< 0.001	0.787^a^	72.40%	96.70%
CD8 + (× 10^9^/L)	0.912	0.830–0.944	< 0.001	1.526^a^	82.80%	95.10%
CD3-CD19 + (×10^9^/L)	0.854	0.754–0.953	< 0.001	0.327^a^	72.40%	93.40%
NK(×10^9^/L)	0.929	0.857–1.000	< 0.001	0.266^a^	82.10%	98.40%
Plasma EBV-DNA load (copies/mL)	0.838	0.686–0.989	< 0.001	47,250.000^b^	77.80%	94.70%
Total blood EBV-DNA load (copies/mL)	0.715	0.578–0.851	0.003	1,660,000.000^b^	64.00%	79.60%
D-Dimer (ug/L)	0.905	0.809–1.000	< 0.001	1721.500^b^	88.90%	90.20%

^a^the data ≤ the value were considered positive, ^b^the data ≥ the value were considered positive

## Discussion

In children and adolescents, the main clinical manifestations of EBV-IM include fever, pharyngitis, superficial lymphadenopathy, hepatosplenomegaly, and skin rash. EBV-IM is primarily transmitted through saliva contact, but it can also spread through blood or sexual transmission, usually accompanied by an increase in peripheral blood heterotypic lymphocytes. HLH is a kind of histiocytosis with reactive hyperplasia of the monocyte-macrophage system. This condition and the resulting clinical syndrome are due mainly to a dysfunction of cytotoxic killer (CTL) and NK cells in which they fail to remove antigen properly, and the resulting sustained antigen stimulation over-activates the mononuclear-macrophage system, producing a large number of inflammatory cytokines. Hemophagocytic syndrome can be divided into primary/familial HLH and secondary HLH, EBV-HLH includes primary HLH induced by EBV and secondary hemophagocytic syndrome driven by EBV, EBV-HLH is the most important type of secondary HLH. The majority of EBV-IM cases are self-limited and mild with a good prognosis. In contrast, most cases of EBV-HLH are severe, involve multiple organs, and are life-threatening [7]. Therefore, the early identification of EBV-HLH by a simple laboratory examination is of great significance.

After a host is infected with EBV, the virus invades the lymphocyte system, inducing the production of a large number of inflammatory factors that subsequently cause enlargement of the liver, spleen, and superficial lymph nodes. The inflammatory factor IL-1, IL-6, TNF-α, among others, acts on the thermoregulatory center, causing a high fever [8]. By inducing immune system factors, EBV infection can damage the functions of various organs, with liver damage being the most common. Rather than being caused by a direct invasion of the liver by the virus, the liver damage is often due to an infiltration of mononuclear cells in the hepatic lobule and portal area [9], free radical hyperactivity in intracellular lipid peroxidation caused by EBV infection, and hepatomegaly caused by B lymphocyte activation [10]. Here, it was found that liver, spleen, and lymph node enlargement all manifested in both EBV-IM and EBV-HLH cases, but the peak fever temperature and duration of fever differed, as did the incidence of liver, spleen and lymph node enlargement. Children with EBV-HLH had a longer duration of pre-hospital fever and overall duration of fever compared with those with EBV-IM; furthermore, elevated levels of alanine aminotransferase, aspartate aminotransferase, lactate dehydrogenase, triglyceride, and ferritin were more obvious in the EBV-HLH cases. The possibility of EBV-HLH should be strongly considered in cases with persistent high fever, significant enlargement of the liver, spleen, and changes in the levels of alanine aminotransferase, aspartate aminotransferase, lactate dehydrogenase, triglyceride, and ferritin.

If the disease progresses further after the initial EBV infection, the accompanying excessive cytokine secretion leads to cytokine storms, such that an immune disorder and uncontrolled inflammatory reactions coexist [11]. In these cases, IFN-γ and TNF-α inhibit the hematopoietic function of bone marrow, and the function of activated macrophages is out of control, together resulting in hemophagocytosis [12]. The inadequacy and deficiency of cellular immunity function after EBV infection are the main causes of severe diseases, such as malignant histiocytosis and lymphoma [13]. The results of this study show that the absolute values of CD3^+^, CD4^+^, CD8^+^, CD3-CD19^+^, and NK cell counts were significantly lower in the EBV-HLH group than in the EBV-IM group, indicating a serious immunodeficiency in the EBV-HLH cases. The CD4^+^/CD8^+^ lymphocyte ratio of the EBV-HLH group was higher than that of the EBV-IM group, indicating that the ability of CD8^+^ lymphocytes to activate and proliferate was lower in the EBV-HLH group than in the EBV-IM group, and, consequently, the virus clearance ability of the EBV-HLH group was weaker [11]. NK cells are important immune cells involved in immune surveillance and early anti-infection, and they can directly kill target cells that are infected by a virus. A decreased amount of NK cells in the peripheral blood and the corresponding decreased ability to clear viruses indicate that the disease may worsen [14], requiring active treatment.

EBV antigen can induce the body to produce specific antibodies, which combine with EBV antigen to form antigen–antibody immune complexes that cause an abnormal proliferation of humoral immunity [15]. Here, the EBV-HLH group had more serious immune defects compared with the EBV-IM group, and the levels of IgA and IgM in peripheral blood of the children in the EBV-HLH group were significantly lower than those in EBV-IM group. Compared with the EBV-IM group, the IgG trended lower in the EBV-HLH group children, but this difference was not statistically significant, indicating that the affected humoral immunity was mainly due to a change of immunoglobulin in the acute phase.

The EBV DNA loads in whole blood and plasma for the EBV-HLH group were significantly higher than those in the EBV-IM group. This finding indicates that EBV was better able to evade the host immune system in the EBV-HLH group, and the cellular and humoral immune functions of the patients in this group were significantly lower, thus allowing the proliferation of EBV throughout the body, further damaging organs and leading to the occurrence of EBV-HLH. The possibility of EBV-HLH should be strongly considered in children with a high EBV DNA load and fever, clearly abnormal liver function, and abnormal levels of lactate dehydrogenase, triglyceride, and ferritin.

In this study, the plasma EBV DNA load in children with EBV-IM was positively correlated with the level of D-Dimer, and the plasma EBV DNA load in children with EBV-HLH was positively correlated with the levels of PT and TT. During EBV infection, monocytes, macrophages, and endothelial cells not only produce inflammatory factors, but also express tissue factor and initiate exogenous coagulation reactions, thereby consuming a large amount of coagulation factors and leading to fibrinolytic hyperactivity and decreased coagulation factor synthesis. The levels of Scr, BUN, UA, TG, and EBV DNA load in children with EBV-HLH were positively correlated, indicating that inflammatory factors affected liver synthesis and secretion as well as kidney function and directly inhibited the level of lipoprotein lipase, causing an increase of triglyceride [16]. Inflammatory factors can invade all organs of the body. Here, the plasma EBV DNA load in children with EBV-HLH was positively correlated with the levels of α-HBDH, LDH, CK, and AST, suggesting that children with EBV-HLH had different degrees of organ damage. Additionally, the plasma EBV DNA load was positively correlated with the CRP level, suggesting that children with EBV-HLH suffer from multiple organ damage and severe immune dysfunction, as well as severe inflammatory reactions.

D-Dimer is a specific degradation product under the action of plasmin after cross-linking of fibrin, and its increased level indicates that the body’s secondary fibrinolytic activity is enhanced, reflecting the body’s hypercoagulable and fibrinolytic state, and can be used for diagnosis Thromboembolic disease. And previous studies have shown that the elevation of D-Dimer is an important independent and persistent risk factor for cardiovascular events and cancer events [17], as well as an early sign of impending MAS in febrile patients with active rheumatism and monitoring indicator for severe infections [18, 19]. In this study, a ROC curve was used to analyze the diagnostic efficiency of various markers for EBV-HLH. It revealed that the diagnostic values of the D-Dimer, IgA, and IgM levels and the CD3^+^, CD4^+^, CD8^+^, CD3-CD19^+^, and NK cell counts were all better than the plasma EBV DNA load and whole blood EBV DNA load, which suggests that the immune function changes after EBV infection are a main factor for the occurrence of EBV-HLH. After EBV infection, perforin and granzyme are released from CD8^+^ and NK cells to kill all kinds of infected or tumor cells. However, the sensitivity and specificity of the IgA and IgM levels as diagnostic markers were low in the EBV-infected children, and the CD3^+^, CD4^+^, CD8^+^, CD3-CD19^+^, and NK cell counts could not be used to distinguish between EBV-IM and EBV-HLH because of their low sensitivity. D-Dimer level was the most valuable indicator for distinguishing between EBV-IM and EBV-HLH in this study. The sensitivity and specificity of the D-Dimer level (cut-off point of 1721.500 μg/L) for use in the diagnosis of EBV-HLH were 88.90 and 90.20%, respectively. However, this study is a small sample study, and the study is limited to 1–3 year-old children, which has certain limitations. Subsequent studies should be further in-depth and expand the sample size. Moreover, not all patients have completed the examinations for immunodeficiency and molecular tests to distinguish between primary and secondary hemophagocytic syndrome, and most patients have progressed rapidly and have not completed the relevant examinations, but the patient had no previous history of repeated infections or immunodeficiency. In this study, it was highly suspected that the clinical manifestations of these children were caused by EB virus infection, thus the serum and/or plasma EBV viral load were checked. But, the cut-off of the serum and/or plasma EBV viral load for EBV infection could not be obtained.

## Conclusions

In summary, the immune function changes after EBV infection are the main factor for the occurrence of EBV-HLH in EBV-infected children, and the D-Dimer level may be a useful indicator for the possibility HLH developing in children with EBV.

## Data Availability

All data is available.
